# Okadaic acid triggers NFκB and STAT3 phosphorylation followed by a release of inflammatory markers in human and mouse endothelial cells

**DOI:** 10.1007/s00204-026-04320-3

**Published:** 2026-02-17

**Authors:** Klara Nyback, Amparo Alfonso, Rebeca Alvariño, Toshiyuki Suzuki, Ryuichi Watanabe, Hajime Uchida, Mercedes R. Vieytes, Luis M. Botana

**Affiliations:** 1 Departamento de Farmacología, Facultad de Veterinaria, IDIS, Universidad de Santiago de Compostela, Lugo, 27002 España; 2 Departamento de Fisiología, Facultad de Veterinaria, IDIS, Universidad de Santiago de Compostela, Lugo, 27002 España; 3 Kitasato University School of Marine Biosciences, Applied Marine Biological Chemistry, Laboratory of Food Chemistry, Kitasato University , 1-15-1 Kitazato, Minami-ku, Sagamihara, Kanagawa Prefecture 252-0373 Japan; 4 Fisheries technology institute, Japan Fisheries Research and Education Agency, 2-12-4 Fukuura, Kanazawa-ku, Yokohama, 236-8648 Japan

**Keywords:** Okadaic acid, Inflammation, Endothelial cells, NFkB, STAT3

## Abstract

Okadaic acid (OA) is a lipophilic phycotoxin that causes acute diarrhoea when ingested. OA is an inhibitor of protein phosphatase 2 A, but the mechanism of toxicity behind the diarrhoea remains unclear. OA modulated inflammatory markers in epithelial cells, however, the effect on endothelial cells, with a key role in the inflammatory cascade, has not been previously addressed. Therefore, the aim of the present work was to test the effect of OA in human (HMEC-1) and mouse (MS1) endothelial cells. After 3, 6 and 24 h of incubation in the presence of OA (10-1000 nM) cell viability was significantly reduced, showing a higher effect on human cells with half inhibitory concentrations (IC_50_) in HMEC-1 cells five times lower than in mouse cells. Furthermore, when cells were treated with OA, significant amounts of the proinflammatory mediators ROS, CD147, IL-6 and monocyte chemoattractant protein 1 (MCP-1) were detected. Some of these effects were observed only in HMEC-1 cells and around three hours earlier, pointing again to a higher sensitivity in human models. Finally, OA triggered phosphorylation of NFκB at 100 nM after 3 and 6 h of treatment, while the signal transducer and activator of transcription 3 (STAT3) was increased after 3 h but decreased after 6 h in both cell lines. Altogether, these data suggest that the toxic effect of OA in endothelial cells could be related with the activation of the inflammatory cascade.

## Introduction

Okadaic acid (OA) is a lipophilic phycotoxin that can be found in mussels and other shellfish. OA is part of the group diarrhoetic shellfish toxins (DSTs), along with dinophysistoxin 1 and − 2 (DTX1 and DTX2)(EFSA 2008). DSTs are the most frequently reported cause of phycotoxin poisonings in Europe and are monitored all over the world (Bresnan et al. 2021). As the name suggests, the main symptoms of these toxins are gastrointestinal, such as diarrhoea, stomach aches and nausea(EFSA 2008). Symptoms tend to appear fast, often within a few hours after ingestion. The safety limit for DSTs in Europe, set by the European Commission (EC 853/2004) is 160 µg OA equivalents per kg shellfish meat(EU 2004). When transforming the amount of DTX1 and − 2 into OA equivalents, toxicity equivalency factors (TEFs) of 1 and 0.6 are used, respectively(WHO 2016). This would indicate that DTX1 has the same toxicity as OA, while DTX2 is less toxic. These factors are based on lethality after intraperitoneal injection in mice, although lethality in humans is not a major concern in OA poisoning(EFSA 2008; WHO 2016). A study compiling phycotoxin poisoning events on the Atlantic coast of Europe from the late 1970s until 2019 based on data in literature and the Harmful Algal Event Database (HAEDAT) reported no cases of DST poisoning ending in human fatalities(Bresnan et al. 2021).

The mechanism of action of OA is the inhibition of protein phosphatase 2 A (PP2A)(EFSA 2008; Munday 2013). Although the mechanism of action is established, the mechanism of toxicity causing diarrhoea is still unclear, increasing the complexity to define TEFs(Louzao et al. 2022; WHO 2016). Another complicating factor is that OA has different effects in different cell lines. For example, the LD_50_ of OA was reported as 38.37 nM in neuroblastoma SH-SY5Y cells, while in differentiated colon carcinoma cells no decline in cell viability after treatment with 2000 nM OA was observed (Louzao et al. 2015). This suggests that the choice of cell line is crucial when testing the effects of OA, and the lack of clear mechanism of toxicity or target organ complicates the choice.

After DST intoxication, intestinal symptoms are quite similar to other gastrointestinal disorders characterised by an inflammation of the intestine triggered by damage to the epithelial cells, which induces symptoms like digestive issues, diarrhoea and abdominal pain (Britzen-Laurent et al. 2023; Saez et al. 2023). In mice, OA has been shown to cause disruption of the epithelial cell layer, swelling of the mitochondria, diarrhoea, swollen stomach and blood in the intestinal lumen (Abal et al. 2018; Costas et al. 2022). A key event in intestinal inflammation is the activation of the microvascular endothelial cells (Britzen-Laurent et al. 2023). Endothelial cells form the walls of the capillaries located in the connective tissue (*lamina propria*) right under the epithelial cell layer (Britzen-Laurent et al. 2023). The endothelium is activated by cytokines released early in the inflammatory response, which leads to the phosphorylation of Nuclear Factor kappa B (NFκB) (Britzen-Laurent et al. 2023; Wu et al. 2021). NFκB is a transcription factor regulating the production of several pro-inflammatory cytokines, interleukins and cell adhesion molecules (CAMs). Therefore, it is an important mediator in the inflammatory response (Atreya et al. 2008; Britzen-Laurent et al. 2023). To recruit immune cells, microvascular endothelial cells express CAMs like vascular cell adhesion molecule 1 (VCAM-1), monocyte chemoattractant protein 1 (MCP-1) and E-selectin on the cell surface at the site of inflammation. CAMs then bind to immune cells and facilitate their migration into the lamina propria (Britzen-Laurent et al. 2023; Saez et al. 2023; Wu et al. 2021). Endothelial cells release cytokines, such as IL-6 and the receptor CD147, which further activate the inflammatory cascade (Atreya et al. 2008; Britzen-Laurent et al. 2023). IL-6 can induce phosphorylation of signal transducer and activator of transcription 3 (STAT3), as well as trigger further cytokine release, and CD147 can induce NFκB and increase cytokine secretion (Morris et al. 2018; Saez et al. 2023; Wu et al. 2021). Endothelial cells also release reactive oxygen species (ROS) early in the inflammatory response, which can cause cell damage and increase the levels of CAMs (Saez et al. 2023; Tanida et al. 2011).

Recent in vitro studies have proposed that OA could cause an inflammatory response (del Campo et al. 2017; Ferron et al. 2014; Reale et al. 2021; Wuerger et al. 2023). For example, NFκB phosphorylation and expression of pro-inflammatory cytokines have been detected in several cell lines treated with OA (Reale et al. 2021; Wuerger et al. 2023). In this context, the aim of this work was to test the effect of OA in the activation of the inflammatory response in endothelial cells. To address differences between humans and mice, which are relevant as the regulatory safety limits are based on data from mice, cells from both species were used. These cell lines have not previously been used to study the toxic and inflammatory effects of OA, despite their role in intestinal inflammation.

## Materials and methods

### Chemicals and solutions

OA was provided by Drs T. Suzuki, R. Watanabe and H. Uchida. Pierce Protease Inhibitor Mini Tablets EDTA free and Pierce Phosphatase Inhibitor Mini Tablets EDTA free were purchased from Thermo Fisher Scientific (Madrid, Spain). Anti-phospho-NFκB antibody, anti-NFκB antibody, anti-phospho-STAT3 antibody and anti-STAT3 antibody were purchased from Abcam (Cambridge, UK). Polyacrylamide Mini PROTEAN TGX Gels and Precision Plus Protein Standard Kaleidoscope were purchased from BioRad (Madrid, Spain). Anti-β-Actin, 3-(4,5-dimethyl thiazol-2-yl)-2,5-diphenyl tetrazolium bromide (MTT), saponin, lipopolysaccharide (LPS) and the rest of the reagents used were purchased from Merck (Madrid, Spain). Hypotonic buffer used to obtain cytosolic lysates was composed of 20 mM Tris-HCl (pH 7.4), 10 mM NaCl and 3 mM MgCl_2_ and a phosphatase/proteinase inhibitor cocktail. Locke’s buffer contains (in mM): 154 NaCl, 5.6 KCl, 1.3 CaCl_2_, 1 MgCl_2_, 3.6 NaHCO_3_, 5 Glucose and 10 HEPES. PBS was composed of (in mM): 137 NaCl, 8.2 Na_2_HPO_3_, 1.5 KH_2_PO_3_ and 3.2 KCl.

### Cell culture

Human dermal microvascular endothelial cell line (HMEC-1) was obtained from American Type Culture Collection (ATCC), number CRL 3243. Cells were cultured in MCBD131 media, supplemented with 10% foetal bovine serum (FBS), 5% Glutamax, epithelial growth factor (10 ng/L), 1% penicillin and streptomycin and hydrocortisone (1 µg/L). Mouse pancreatic endothelial cell line (MS1) was also purchased from ATCC, number CRL 2279. MS1 were cultured in Dulbecco’s Modified Eagle Medium with high glucose (4.5 g/L), supplemented with 5% FBS and 1% penicillin and streptomycin. The cells were maintained in an incubator at 37 °C with 5% CO_2_ and 95% air in a humid environment and subcultured every week using trypsin/EDTA. All the reagents were purchased from Thermo Fisher Scientific.

### Cell viability and cytotoxicity assays

Cell viability was assessed with the MTT assay, as previously described (Alvariño et al. 2022). Cells were seeded at 2 × 10^4^ cells/well in a 96-well plate and incubated overnight. Cells were then treated with 10, 50, 100, 500 and 1000 nM OA, or saponin (1 mg/mL) as a death control, then incubated for either 3, 6–24 h. After incubation, cells were washed with Locke’s buffer and incubated with MTT (500 µg/mL) for 1 h at 300 rpm and 37 °C. Next, cells were disaggregated using 5% SDS. Absorbance was measured at 595 nm in a spectrophotometric plate reader.

The effect of OA in cytotoxicity was analysed using the CYQUANT LDH Cytotoxicity Assay Kit (Thermo Fisher Scientific) following manufacturer’s instructions. Cells were seeded and treated as described above, 50 µL of supernatants were transferred to another plate and LDH release was determined. Absorbance was measured at 490 nm with a spectrophotometric plate reader.

All experiments were repeated three independent times.

### Quantification of IL-6

Cells were seeded in a 96-well plate (2 × 10^4^ cells/well) and treated with 10, 50, 100, 500 and 1000 nM OA for 3, 6 and 24 h. LPS (500 ng/mL) was used as a positive control of inflammation. Three individual experiments were performed for each time point. Supernatants were collected and stored in -80 °C. IL-6 was quantified using Human IL-6 Uncoated ELISA kit and Mouse IL-6 Uncoated ELISA kit (Thermo Fisher Scientific) in HMEC-1 and MS1 cells, respectively, following manufacturer’s instructions. The absorbance was read at 450 nm in a spectrophotometric plate reader.

### Measurement of IL-1β, E-selectin, VCAM-1, MCP-1 and CD147

IL-1β, E-selectin, VCAM-1, MCP-1 and CD147 were measured in HMEC-1 supernatant with the Human ProcartaPlex Mix&Match 5-plex kit (Thermo Fisher Scientific), following manufacturer’s instructions. HMEC-1 cells were seeded in a 12-well plate (15 × 10^4^ cells/well), treated with 10, 50 and 100 nM OA and incubated for 6–24 h. LPS (500 ng/mL) was used as a positive control and untreated cells as control. The supernatants were collected and stored in -80 °C until analysis. Fluorescence was measured using the Luminex xMAP INTELLIFLEX instrument with the xMAP INTELLIFLEX Software. MCP-1 and CD147 were also measured in MS1 cells supernatants using EMMPRIN (CD147) Mouse SimpleStep ELISA Kit (Abcam) and Mouse MCP-1 ELISA Kit (Thermo Fisher Scientific) according to the manufacturers’ instructions.

All experiments were repeated three independent times.

### Measurement of reactive oxygen species levels

The release of ROS was measured using carboxy-H_2_-DCFDA dye(Castedo et al. 2025). Cells were seeded in a 96-well plate (2 × 10^4^ cells/well) and treated with 10, 50, 100, 500 and 1000 nM OA for 6 h. Hydrogen peroxide (150 µM) was used as a positive control. Then cells were washed twice with serum-free media and 20 µM carboxy-H_2_-DCFDA was added for 1 h, at 37 °C and 300 rpm. Then, PBS was added for 30 min, at 37 °C and 300 rpm. Fluorescence was measured at a 488/535 nm in a plate reader. Three individual experiments were performed in triplicate.

### Western blotting

Cells were seeded at 15 × 10^4^ cells/well in 12-well plates and treated with 100 nM OA for 3 and 6 h. LPS (500 ng/mL) was used as positive control. Cells were washed with PBS and 100 µL hypotonic buffer were added. The cells were scraped and incubated for 15 min on ice and 5 µL of Triton X-100 (5%) were added to each sample. Next, samples were centrifuged at 3000 rpm for 10 min at 4°C and the supernatant was collected as the cytosolic fraction. Protein content was quantified with Direct Detect system (Merck).

Electrophoresis was performed in 4–20% polyacrylamide gels, with 15 µg protein from the cytosolic lysates. The protein weight was determined using Precision Plus Protein Standard Kaleidoscope molecular weight marker. Protein transfer was performed in the semi-dry Transblot system. Membrane blockage and antibody incubation were carried out with the Snap i.d. system (Merck), followed by incubation with SuperSignal West Pico PLUS Chemiluminescent Substrate or Supersignal West Femto Maximum Sensitivity Substrate (Thermo Fisher Scientific). Protein bands were analysed with the Diversity Gene Snap system and Gene Snap and Gene Tools software (Syngene). Phosphorylated NFκB-p65 was detected with anti-phospho-NFκB p65 (1:1000) and total NFκB-p65 with anti-NFκB p65 (1:1000). STAT3 was quantified with anti-phospho-STAT3 (1:1000) and total STAT3 with anti-STAT3 (1:1000). Protein band intensities were normalised by β-Actin (1:5000). At least three independent experiments were carried out by duplicate.

### Statistical analysis

Results are expressed as mean ± SEM. Statistical comparisons were analysed with ANOVA test and Dunnett’s *post hoc* test using the GraphPad Prism v.8 software. Statistical significance was considered at *p* < 0.05.

## Results

OA’s effect on microvascular endothelial cells has not been targeted before, despite the cells’ important role in the intestinal barrier. Cell viability was first addressed to check possible differences between human and mouse cells and determine a suitable dose range of OA for further testing.

**Fig. 1:**
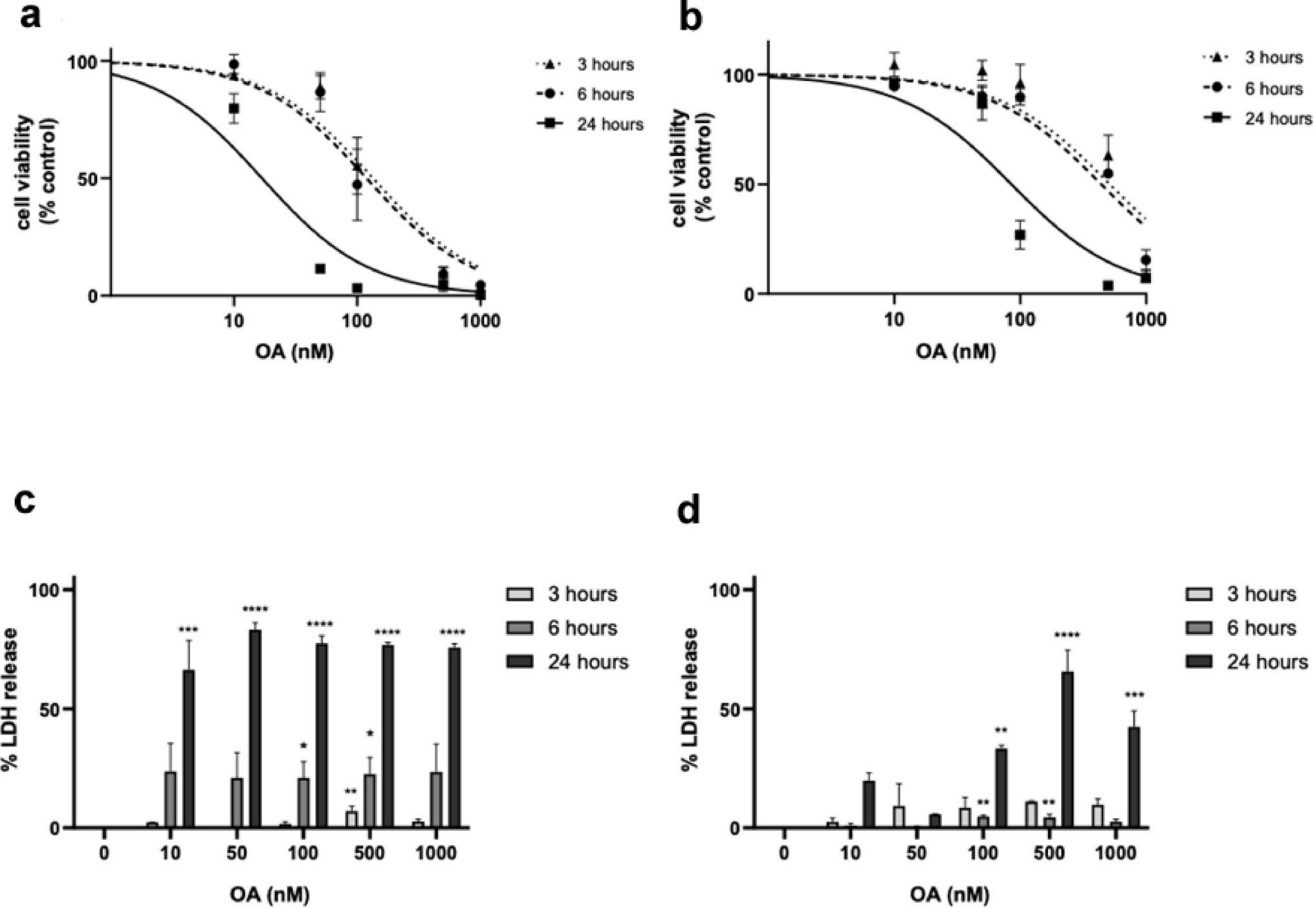
Effect of OA on viability and LDH release in human (HMEC-1) and mouse (MS1) microvascular endothelial cells. **a** Cell viability of HMEC-1 cells treated with OA for 3, 6–24 h. **b** Cell viability of MS1 cells treated with OA for 3, 6–24 h. Results are expressed as percentage of control cells and presented as mean ± SEM of three independent replicates performed by triplicate. **c** LDH release in HMEC-1 cells and d) MS1 cells treated with OA for 3, 6–24 h. Data are expressed as percentage of cell death control and presented as mean ± SEM of three independent replicates performed by duplicate. Statistical differences were assessed by one-way ANOVA followed by Dunnett’s tests (* *p* < 0.05, ** *p* < 0.01, *** *p* < 0.001, **** *p* < 0.0001 compared to untreated control).

 After AO treatment, endothelial cells showed a dose dependent decline in viability (Fig. 1a and b). The cell viability of HMEC-1 cells (Fig. 1a) starts declining earlier than the cell viability of MS1 cells (Fig. 1b). The difference is most apparent at 100 nM OA, when after 3 and 6 h of incubation, human endothelial cells have slightly over 50% viability, while mouse endothelial cells have closer to 80% viability. After 24 h, HMEC-1 cells have 10–15% viability at 100 nM OA and MS1 cells have slightly under 50% compared to control cells. To further assess the difference in cell viability, values for the half inhibitory concentrations (IC_50_) were determined using non-linear regression. As shown in Table 1, IC_50_ in HMEC-1 cells after 3 and 6 h were about four times lower than the IC_50_ in MS1 cells at the same timepoints. The difference is even larger after 24 h, when human cells have an IC_50_ of 16.8 nM and the IC_50_ of MS1 cells is 88.5 nM, which is about five times higher. These results suggest that human endothelial cells are more sensitive to OA than mouse endothelial cells. Then, to assess the cellular toxicity of OA, LDH was measured as a marker of cell death. Human endothelium showed less than 10% cell death after 3 h of incubation, around 20% after 6 h, and over 75% cell death compared to control in all OA concentrations tested after 24 h (Fig. 1c). In comparison, low levels of LDH release were detected in mouse endothelial cells after 3 and 6 h of incubation, the highest level detected was 11% at 500 nM OA after 3 h of incubation (Fig. 1d). After 24 h MS1 cells showed significant levels of LDH at 100, 500 and 1000 nM OA (50%). Overall, a larger portion of the human cells died in comparison to mouse cells after OA treatment.

**Table 1:** Half inhibitory concentration (IC_50_) in HMEC-1 and MS1 based on results from the MTT assay after treatment with 10, 50, 100, 500 and 1000 nM OA for 3, 6 and 24 h.

	IC_50_ 3 h	IC_50_ 6 h	IC_50_ 24 h
HMEC-1	134.4 nM	120.3 nM	16.8 nM
MS1	514.3 nM	440.2 nM	88.5 nM

The next step was to determine if OA could cause an inflammatory reaction in endothelial cells. Inflammatory response is initiated and managed through several mediators, such as cytokines (Wu et al. 2021). IL-6 is a pro-inflammatory cytokine involved in the progression of intestinal inflammation, so its levels were quantified in the supernatant of HMEC-1 and MS1 cells treated with OA.

**Fig. 2:**
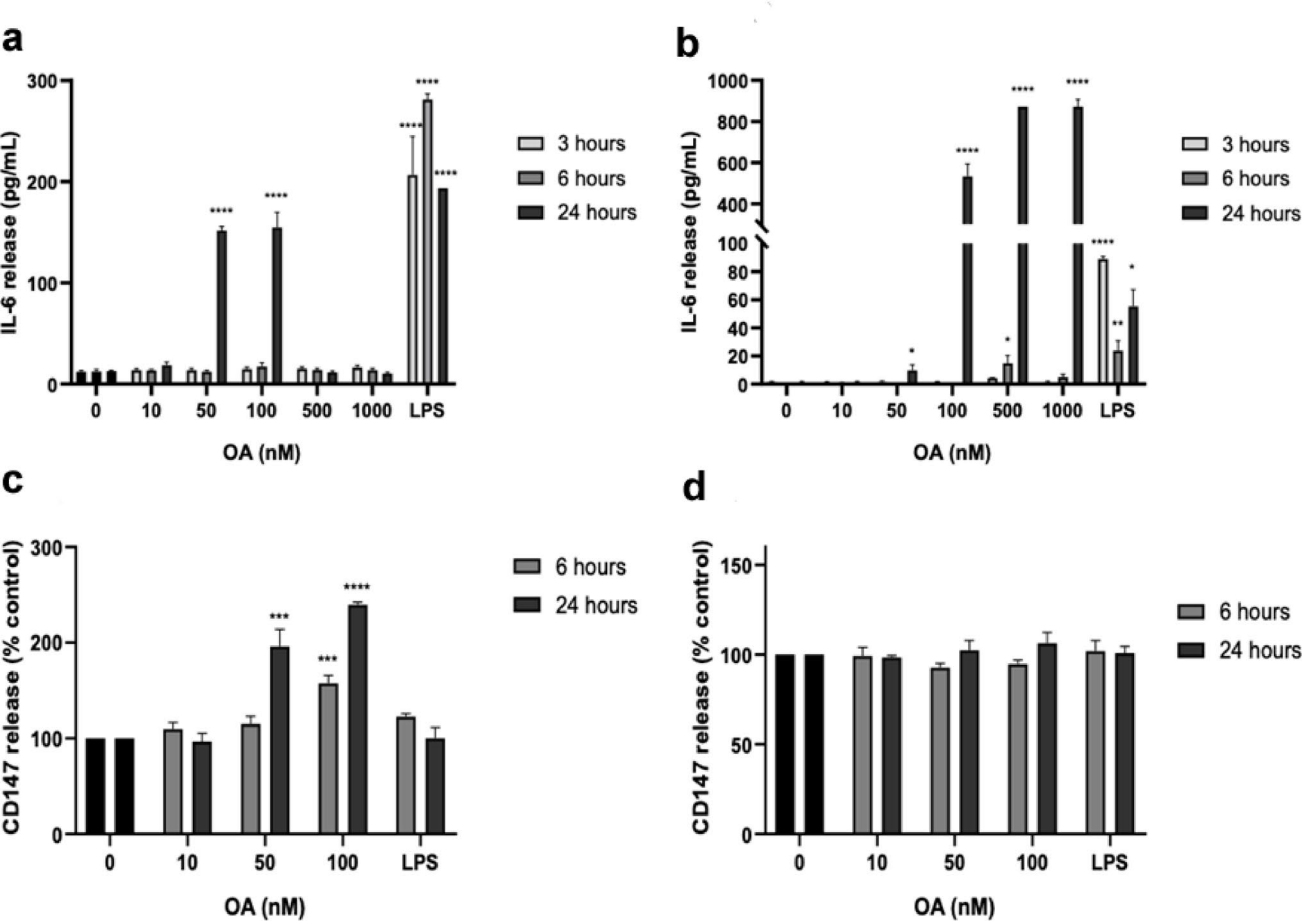
Measurement of IL-6 and CD147 release from human (HMEC-1) and mouse (MS1) microvascular endothelial cells treated with OA. **a** IL-6 release in HMEC-1 and **b** IL-6 release in MS1 after OA treatment. **c** Levels of CD147 receptor measured in HMEC-1. **d** Levels of CD147 receptor measured in MS1. LPS at 500 ng/ml was used as a positive control. Mean ± SEM of three independent replicates performed by duplicate. Statistical differences were assessed by one-way ANOVA followed by Dunnett’s tests (* *p* < 0.05, ** *p* < 0.01, *** *p* < 0.001 **** *p* < 0.0001 compared to untreated control.

As Fig. 2a-b shows, both cell lines showed increased release of IL-6 after 24 h of OA treatment. Human endothelium released 151.6 ± 4.0 pg/mL and 154.9 ± 14.7 pg/mL IL-6 after treatment with 50 and 100 nM OA for 24 h, respectively (Fig. 2a. In MS1 cells, IL-6 reached levels of 533.1 ± 60.2 pg/mL, 872 pg/mL and 871.9 ± 35.8 pg/mL after treatment with 100, 500 and 1000 nM OA for 24 h (Fig. 2b. The lack of IL-6 release in HMEC-1 cells treated with 500 and 1000 nM OA is likely due to the low cell viability of HMEC-1 cells at these concentrations of OA. When treated with OA for 3 and 6 h, release of IL-6 was only detected in MS1 cells treated with 500 nM OA for 6 h (14.9 ± 5.5 pg/mL). The positive control of inflammation, LPS, induced an increased release of IL-6 in both cell lines and at all incubation times. Interestingly, LPS induced the same response than OA after 24 h in HMEC-1 cells (100 nM OA), but not in mouse endothelium (1000 nM OA).

**Fig. 3:**
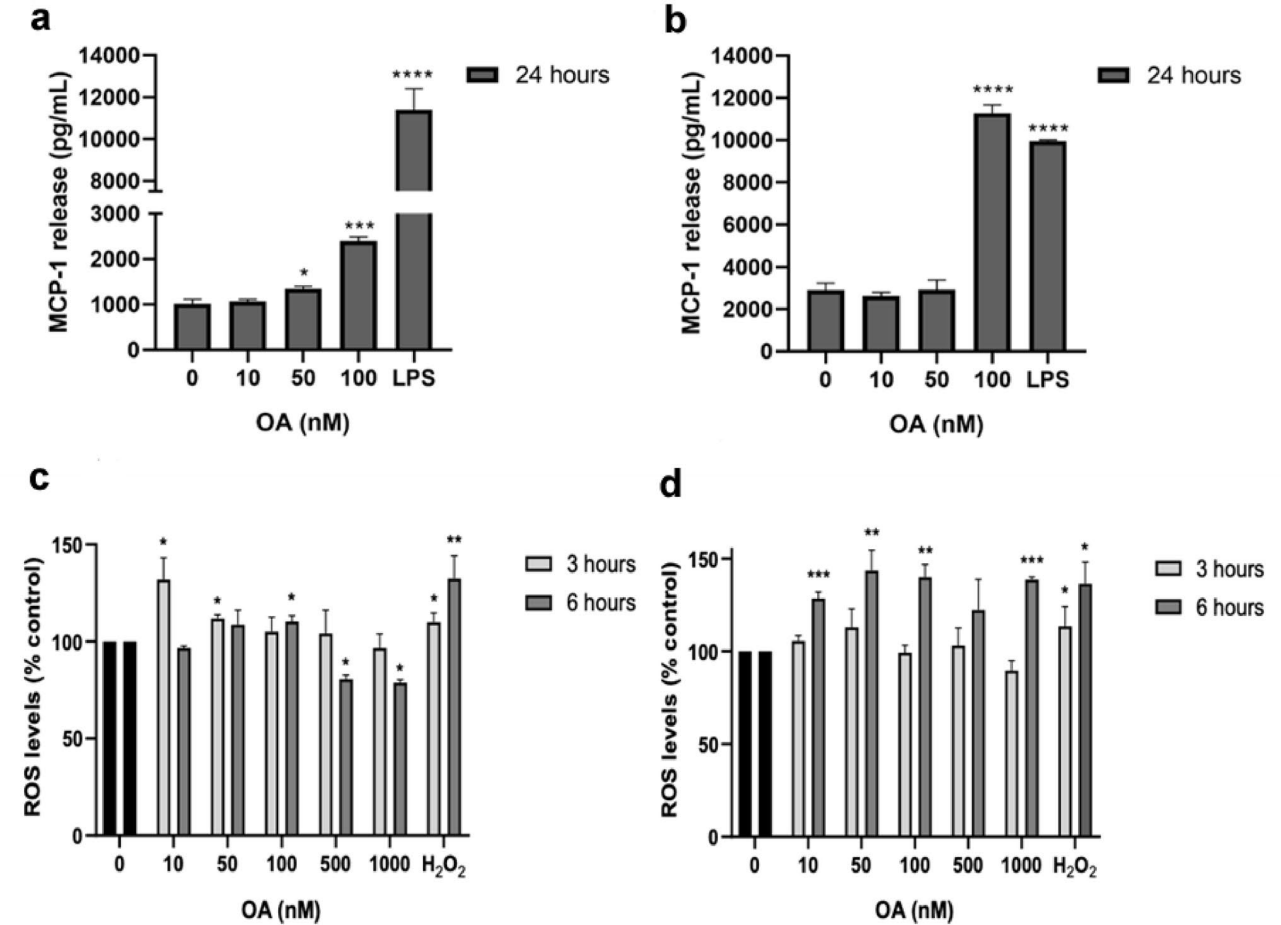
Release of MCP-1 and ROS in human (HMEC-1) and mouse (MS1) cells treated with OA. **a** Levels of MCP-1 release from HMEC-1 cells. **b** Levels of MCP-1 release from MS1 cells. LPS at 500 ng/mL was used as a positive control. **c** ROS levels in HMEC-1 cells and **d** ROS levels in MS1 cells after OA treatment. H_2_O_2_ at 150 µM was used as a positive control. Mean ± SEM from three independent replicates performed in duplicate. Statistical differences were assessed by one-way ANOVA followed by Dunnett’s tests (* *p* < 0.05, *** *p* < 0.001, **** *p* < 0.0001 compared to untreated control).

 Since IL-6 release was increased in the endothelium, other molecules linked to intestinal inflammation were checked. The release of E-selectin, VCAM-1 and MCP-1 was analysed, as they have been proven to facilitate leukocyte infiltration from blood vessels into *lamina propria* in intestinal inflammation (Britzen-Laurent et al. 2023; Tanida et al. 2011). CD147, which is involved in NFκB activation and cytokine release, and IL-1β, which is a pro-inflammatory cytokine usually released in the early stages of inflammation were measured as well (Britzen-Laurent et al. 2023; Xu et al. 2020). These mediators were checked after 6- and 24 h incubation, since IL-6 was detected after 24 h at 50 and 100 nM OA, and 6 h was included to account for a possible earlier reaction. The release of E-selectin, VCAM-1 and IL-1β was not modified after OA treatment (data not shown), while CD147 and MCP-1 levels were increased (Figs. 2c-d and 3a-b). After 24 h, human endothelium showed a dose dependent increase in CD147 (Fig. 2c), reaching levels of 196.2 ± 17.6% and 239.7 ± 2.8% after treatment with 50 and 100 nM OA, respectively. After addition of the same concentrations for 6 h, CD147 levels reached 115.2 ± 7.8% and 157.6 ± 8.4% of control cells, but only 100 nM OA caused a statistically significant increase. In these conditions, no effect on CD147 release was observed in MS1 cells (Fig. 2d, with levels between 92 and 100% (6 h) and 98–107% (24 h) of control cells. Again, this suggests that HMEC-1 cells are more sensitive to OA than MS1 cells.

 In addition, both HMEC-1 and MS1 cells released a significant amount of MCP-1 when treated with 100 nM OA (2399 ± 85.8 pg/mL and 11274.6 ± 317.3 pg/mL, respectively) for 24 h. HMEC-1 cells also released significant amounts of MCP-1 when treated with 50 nM OA, 1348.52 ± 51.6 pg/mL, while mouse endothelium did not (Fig. 3a-b). However, after 6 h OA treatment, any MCP-1 release was observed in both cellular lines (data not shown).

 Increased ROS production is a common feature of intestinal inflammation, and excessive amounts of ROS in the *lamina propria* can damage the epithelial cells (Saez et al. 2023). Since OA induce the release of several inflammatory mediators, ROS production was also measured after 3- and 6 h OA treatment, as 24 h resulted in a high cell death in HMEC-1 cells. After 3 h of incubation, HMEC-1 cells showed a significantly increased ROS levels after incubation with 10 and 50 nM OA (131.9 ± 11,2% and 111.8 ± 2.1%, respectively) (Fig. 3c). At the same time, no change in ROS release from mouse endothelial cells was detected (Fig. 3d). On the other hand, ROS levels in human cells decreased at 500 and 1000 nM OA after 6 h, probably due to cell death. In mouse endothelium, ROS production was increased after 6 h, with levels between 128.4 ± 3.7% and 139.9 ± 6.9% of control cells after treatment with 10 to 1000 nM OA (Fig. 3d). In that sense, similar ROS levels were observed in both cell lines after treatment with positive control hydrogen peroxide.

**Fig. 4:**
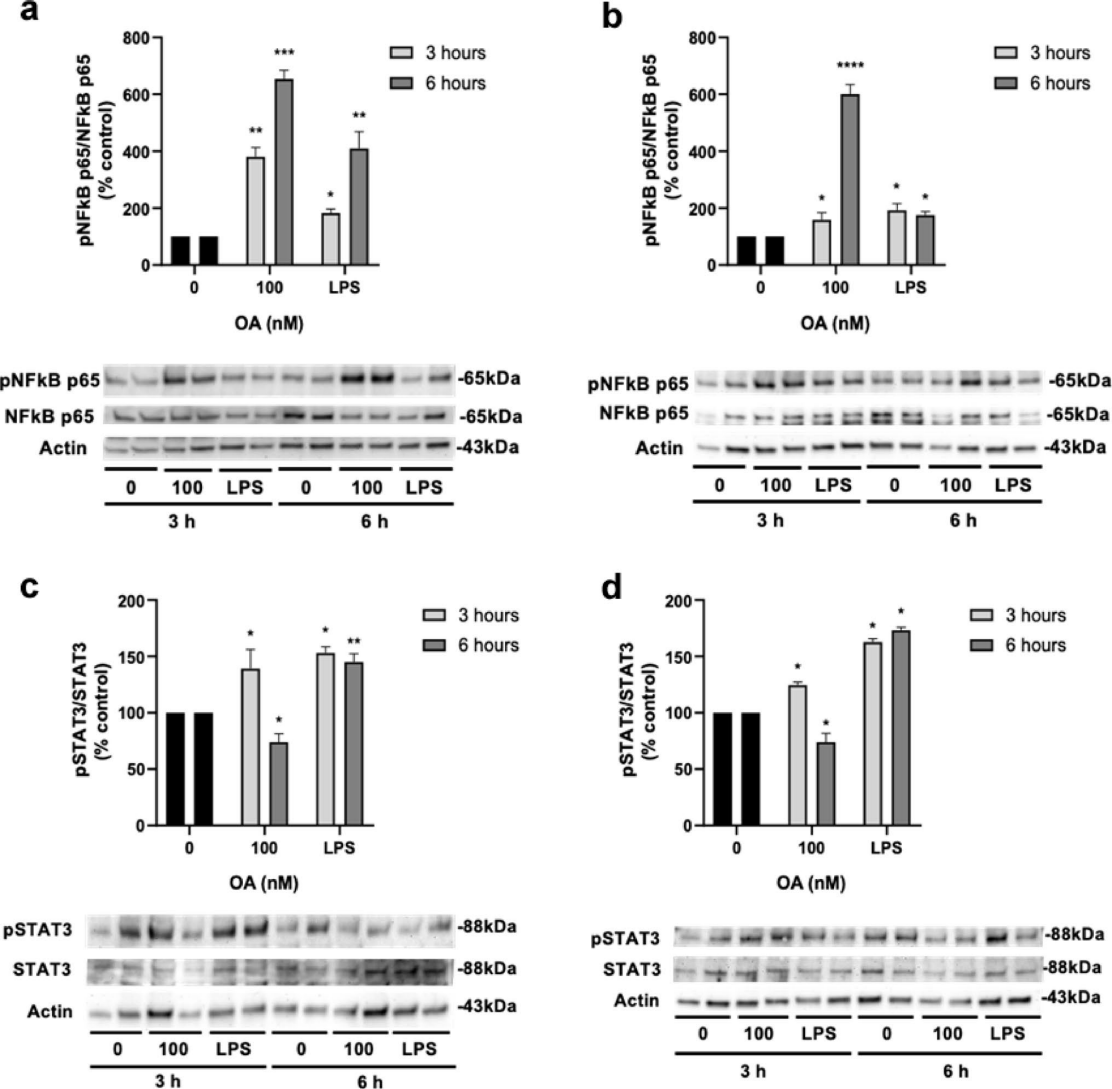
Analysis of NFκB and STAT3 activation in human (HMEC-1) and mouse (MS1) microvascular endothelial cells after treatment with OA. **a** Activation of NFκB-p65 in HMEC-1 cells and **b** activation of NFκB-p65 in MS1 cells. **c** Activation of STAT3 in HMEC-1 and **d** activation of STAT3 in MS1 cells. Data are present as ratio of phosphorylated and total protein and normalized by actin. Cells were incubated with 100 nM OA for 3 and 6 h. LPS 500 ng/mL was used as positive control. Mean ± SEM from three independent replicates performed in duplicate. Statistical differences were assessed by one-way ANOVA followed by Dunnett’s tests (* *p* < 0.05, ** *p* < 0.01, *** *p* < 0.001, **** *p* < 0.0001 compared to untreated control).

 In view of the results obtained in the endothelial cells, the next step was to measure NFκB activation. NFκB is considered the master mediator of inflammation, since it works as a transcription factor for several cytokines and other pro-inflammatory mediators (Atreya et al. 2008; Laurindo et al. 2023). To determine NFκB translocation to the nucleus, phosphorylated (pNFκB-p65) and total NFκB-p65 (NFκB-p65) were quantified in both cell lines. As NFκB activation is involved in the initiation of the inflammatory response in the endothelium, 3 and 6 h were chosen as incubation times (Britzen-Laurent et al. 2023). The concentration of OA tested was 100 nM since it triggered a release of inflammatory markers in HMEC-1 cells without high toxic effect. In human endothelium, treatment with 100 nM OA caused an increase in the levels of NFκB phosphorylation, 380.3 ± 32.2% and 654.2 ± 30.4% after 3 and 6 h (Fig. 4a, respectively. In MS1 cells, the levels of NFκB activation were 159.3 ± 24.3% and 600.8 ± 33.5% after 3 and 6 h, respectively (Fig. 4b). The positive control LPS also induced a statistically significant increase of NFκB activation in both cell lines.

Then, the expression of STAT3, another transcription factor associated with intestinal inflammation, was analysed. STAT3 is also activated by phosphorylation, hence phosphorylated and total STAT3 (pSTAT3 and STAT3) were quantified in the cytosolic fraction (Fig. 4c-d). Both cell lines showed the same patterns of STAT3 phosphorylation after treatment with 100 nM OA: a significant increase after 3 h, followed by a decrease after 6 h. In HMEC-1 cells, the levels of STAT3 activation reached 139.2 ± 17%, and a decrease of 73.9 ± 7.53% was observed after 6 h (Fig. 4c). In mouse cells, STAT3 activation levels reached a 124.6 ± 2.6% after 3 h and decreased to 74 ± 7.76% after 6 h (Fig. 4d). LPS treatment did also result in an increase in STAT3 activation, but the levels remained the same after 6 h, suggesting that the pathways of STAT3 activation were different for OA and LPS. Therefore, these results support the hypothesis that OA can cause an inflammatory reaction in the endothelium.

## Discussion

 The current European regulatory limit for DSTs is 160 µg OA equivalents per kg shellfish meat, which corresponds to the LD_50_ of OA in mice after intraperitoneal injection(EFSA 2008). There are a couple of potential issues with this limit, for example that death is not a common symptom of acute DST poisoning in humans and that mouse toxicity studies using oral administration instead of intraperitoneal yield different results (Abal et al. 2018; Bresnan et al. 2021). In vitro testing of PP2A inhibition has been used to compare the toxicity of the different DSPs, but the lack of understanding of the mechanism of toxicity makes it less useful for setting a safety limit (Ikehara et al. 2021). As mentioned before, the effects of OA are cell line dependent, and without knowing the target organ, it is difficult to argue that the rate of PP2A inhibition in a certain cell line is accurately describing the events of an OA poisoning (Ferron et al. 2014; Louzao et al. 2015; Souid-Mensi et al. 2008). Furthermore, it has not been established that PP2A inhibition alone is the trigger for OA induced diarrhoea, meaning that a regulatory limit only based on PP2A inhibition may not be accurate if there are other crucial factors involved(Munday 2013). The present study focuses on the different effect of OA between mice and humans, as the current regulatory limit is set under the assumption that they are almost comparable. Ingesting DSTs induces diarrhoea in both humans and mice and although that is not the basis of the regulatory limit, it implies that their reactions to the toxin are similar (Costas et al. 2022; Yasumoto et al. 1978). However, the present study indicates that there is a difference in the sensitivity to OA between human and mouse endothelium. The role of endothelial cells in OA intoxication does need further clarification, but it is established that OA, a lipophilic, usually ingested toxin, can pass through the intestinal epithelial cells, meaning that it could reach the endothelium as well (Ehlers et al. 2011; Reale et al. 2021).

 Although the role of endothelium in OA-induced diarrhoea is not fully understood, the function of these cells in inflammatory diseases has been well established (Britzen-Laurent et al. 2023). As mentioned earlier, studies on cell lines connected to the intestinal barrier have shown that exposure to OA can induce a release of pro-inflammatory cytokines (del Campo et al. 2017; Reale et al. 2021). Therefore, it is plausible that an inflammatory reaction to OA in the intestine could be driven by endothelial cells as well. An inflammatory reaction in the endothelium is triggered by the activation of NFκB, which was observed in OA treated cells from both human and mouse in the present study. OA can activate NFκB in other cell lines too, which can be explained by its function as a PP2A inhibitor (Reale et al. 2021; Wuerger et al. 2023). As described earlier, NFκB is activated through phosphorylation, since the NFκB complex is otherwise bound to the IκB protein in the cytoplasm (Mitchell et al. 2016). The function of PP2A in this process is to dephosphorylate NFκB to regulate its activity and avoid hyperphosphorylation (Yang et al. 2001). In that sense, inhibiting PP2A both increases NFκB activation and decreases the normal negative feedback loop of dephosphorylation. On the other hand, it is possible that NFκB is activated by CD147 in addition to OA. CD147 has been shown to induce NFκB phosphorylation in intestinal cells (Xu et al. 2020). In both the present study and other studies on intestinal inflammation, the inflammatory response increased CD147 expression (Wang et al. 2020; Xu et al. 2020). Furthermore, an increase in CD147 levels and activation of NFκB after 6 h at 100 nM OA in human endothelial cells is showed in the present work. However, no change in CD147 expression was observed in mouse endothelium, although NFκB phosphorylation was still increased after 6 h.

 STAT3 is phosphorylated through the JAK/STAT pathway, which is activated when a cytokine binds to the cytokine receptor on the cell surface attached to the Janus kinase (JAK) complex inside the cell, triggering the phosphorylation and release of STAT by JAK (Morris et al. 2018). For STAT3, IL-6 is the main cytokine initiating phosphorylation (Gao et al. 2018; Morris et al. 2018), and its transcription is regulated by NFκB (Atreya et al. 2008). In this sense, it has been proposed that the link between OA and STAT3 is the activation of NFκB due to PP2A inhibition leading to an increased IL-6 release, and subsequently an increased STAT3 activation (Wuerger et al. 2023). However, it is unclear if STAT3 phosphorylation itself is dependent on PP2A or not. A study on T-cells showed that OA-treatment did increase STAT3 activation (Woetmann et al. 1999). In the present study, IL-6 release was significantly increased after 24 h of incubation, although STAT3 phosphorylation increased already after 3 h. This could indicate the involvement of PP2A inhibition or the presence of another cytokine binding to the receptor. But also, IL-6 can be released and attached to the membrane receptor JAK and therefore it was not detected in the extracellular media. STAT3 phosphorylation was downregulated after 6 h of OA-treatment in both cellular lines. This indicates that the mechanism behind dephosphorylation of STAT3 is different from NFκB. It is well established that STAT3 initiates the transcription of suppressor of cytokine signalling 3 (SOCS3), whose main function is to regulate STAT3 phosphorylation (Morris et al. 2018; Suzuki et al. 2001). SOCS3 binds to the JAK complex, inhibiting phosphorylation regardless of cytokine binding (Martino et al. 2022; Morris et al. 2018). The decrease in STAT3 activation after 6 h of OA treatment could therefore represent a natural negative feedback loop.

 One of the most important functions of endothelial cells in inflammation is to express adhesion molecules on the surface to recruit immune cells such as leukocytes to the site. According to literature, NFκB activation is necessary for the expression of VCAM-1, E-selectin and MCP-1 (Denk et al. 2001). In this work, an increase in extracellular MCP-1, but not VCAM-1 or E-selectin was observed in both cell lines in response to treatment with OA. However, CAMs are normally expressed on the cell surface and not released from the cell unless there is an excess of CAMs produced inside the cell. Therefore, the detection of extracellular MCP-1 and CD147 in the present study does indicate a high intracellular level as well, but the lack of extracellular VCAM-1 and E-selectin does not prove an absence inside the cell, only a lack of excess.

ROS release is an early sign of the inflammatory response and can cause cell damage and increase CAM expression on the cell surface (Saez et al. 2023; Tanida et al. 2011). In the present work, HMEC-1 cells clearly released ROS earlier than MS1-cells, suggesting a faster inflammatory reaction. ROS release did not follow the same pattern as the release of MCP-1 and IL-6, which were released after 24 h and treatment with 50 and 100 nM OA in human and mouse cells, respectively. Instead, ROS release was increased already after treatment with 10 nM OA in both cell lines, but 3 h earlier in human than in mouse cells.

 In summary, there are several differences in response to OA in HMEC-1 and MS1 cells suggesting that the human cells are more sensitive, but in both lines OA triggers the inflammatory cascade. IC_50_ in HMEC-1 cells are around five times lower than in MS1 cells after OA treatment. In terms of inflammatory markers, human cells released high amounts of ROS three hours earlier than mouse cells, and lower amounts of OA are necessary to initiate the release of cytokines and adhesion molecules. Although both cell lines showed an inflammatory response to OA, the pattern of decreasing cell viability and the progression of the inflammatory cascade are not directly comparable between human and mouse endothelia. Therefore, these data support that OA toxicity could be meditated by the activation of the inflammatory cascade.

## Data Availability

All data generated or analysed during this study are included in this published article.
